# Bipolar disorder in an evolutionary framework: rethinking adaptation, vulnerability, and stigma

**DOI:** 10.3389/fpsyt.2026.1764726

**Published:** 2026-03-25

**Authors:** Massimo Tusconi, Serdar M. Dursun, Giulia Cossu, Mauro Giovanni Carta

**Affiliations:** 1 Center for Liaison Psychiatry and Psychosomatics, University Hospital Of Cagliari, Cagliari, Italy; 2 Neurochemical Research Unit, Department of Psychiatry, University of Alberta, Edmonton, AB, Canada; 3 Department of Medical Sciences and Public Health, University of Cagliari, Cagliari, Italy

**Keywords:** BD, bipolar disorder, creativity, exploration, high energy, leadership, resilience, stigma

## Introduction

Bipolar Disorder (BD) is one of the most severe mental disorders and one of the top 20 causes of severe impairment in everyday life (1). This disorder is characterized in its most severe form, Type I Bipolar Disorder, by alterations concerning episodes of euphoric mood and irritability, with an exaggeratedly optimistic view of the self, acceleration of ideas, increased energy, and markedly reduced need for sleep, configured as episodes of mania (2). Importantly, bipolar disorder is now recognized as a progressive illness. Over time and with the accumulation of affective episodes, individuals may experience worsening functional outcomes and cognitive decline, reflecting an underlying biological progression of the disease. This conceptualization has been formalized through clinical and biological proposed staging models, which suggest that earlier intervention may mitigate the long-term neuroprogressive trajectory of the disorder (3–6). Such aspects may occur with or without depressive episodes, with reduced interest in various behavioral areas, decreased mood orientation, sleep and appetite disturbances, and a negative view of self, the world, and the future (7). Repeated mood episodes are associated with worsening functional outcomes, increased disability, and greater treatment complexity over time, highlighting the progressive clinical burden of bipolar disorder (8, 9). Considering the high burden of stigma associated with bipolar disorder, exploring its potential adaptive aspects may contribute not only to a deeper scientific understanding but also to reshaping public and clinical perceptions of the disorder. Highlighting the evolutionary value of specific traits, such as increased energy, creativity, and resilience, can counteract deficit-focused narratives and support efforts to reduce stigma. It is important to clarify from the outset that the evolutionary perspective discussed in this article is not intended to replace established clinical, neurobiological, or psychosocial models of bipolar disorder. Rather, it should be understood as a complementary interpretative framework that may help contextualize certain temperamental traits associated with the bipolar spectrum within broader evolutionary and adaptive processes. In this sense, the evolutionary hypothesis proposed here is primarily heuristic and aims to stimulate interdisciplinary reflection on vulnerability, resilience, and stigma in bipolar disorder, rather than to provide a deterministic etiological explanation.

Episodes of everyday mood may be detectable even for remarkably extended periods (10). Subthreshold mood fluctuations and temperamental traits associated with the bipolar spectrum may persist over extended periods and can be observed independently of acute manic or depressive episodes. Delusions or beliefs that cannot be shared and are resistant to criticism may be present in both the depressive and manic phases, placing them in differential diagnosis with a psychotic onset (11). In the form called Bipolar II Disorder, depressive episodes and less severe manic episodes called hypomania are present; delusions are absent in this form (12, 13). Although seemingly less severe, the type II form can be challenging to recognize (14). It thus can also cause severe impairment of activities and enactment of socially inappropriate behaviors not identified as consequences of a pathological condition (15).

A significant delay in the proper diagnosis of bipolar disorder is often observed. While this is partly due to the predominance of depressive episodes that mask the cyclic nature of the illness, it also stems from the fact that certain hypomanic behaviors, such as heightened performance, social charm, increased productivity, and energy, may be perceived as positive traits by both patients and their social environments, thus delaying clinical recognition and intervention (16). Environmental and contextual factors, including modern psychosocial stressors and treatment exposures, may interact with underlying vulnerability and influence the clinical course and complexity of bipolar disorder (17).

Epidemiological surveys in several countries worldwide found the estimated lifetime prevalence of BD in adults to be 2 to 4 percent of the community (18). In the US National Comorbidity Survey Replication, conducted among 9,282 adults aged 18 and older, 83% of people identified as having a diagnosis of BD reported suffering from severe impairment (19).

Family, twin, adoption surveys and molecular studies using several approaches, including new procedures such as Whole Genome Sequencing (WGS), have suggested that genetic factors could significantly contribute to the risk of BD, with heritability estimates of up to 85% (20). First-degree relatives were found to have nine times the risk for illness compared with the general population (21). However, despite strong evidence of high heritability, no individual genetic variant has been conclusively identified as the sole factor associated with the disorder, preventing precise risk prediction (22) and allowing accuracy in identifying risk conditions (22–25).

Genetic linkage research has implicated numerous genomic regions segregating with the disorder in families. Several candidate genes were also identified with evidence of association with BD (26). In a recent genome-wide association (GWA) study evaluating millions of variants across the genome in individuals with BD compared with healthy controls (around 30,000 cases against over 150,000 controls), the authors identified 30 distinct genomic regions (27). One study found a genetic variant, RS1006737, which is enumerated in the literature in association with BD, in well-adjusted older adults without BD but with marked hyperactivity (28, 29).

Several of the identified candidate genes were found to be consistent with current knowledge on the pathophysiology of BD as voltage-gated calcium channels (CACNA1C) (30) synaptic components (e.g., ANK3) and neurotransmitter receptors (e.g., GRIN2A) (31, 32). However, it should be emphasized that while associated variants collectively explain only 4% of disease susceptibility, the combined polygenic signal of common variants accounts for 25% of the genetic variance in BD. Furthermore, 70% of this variance is shared with schizophrenia (33). From an evolutionary perspective, the highly polygenic architecture of bipolar disorder may also be interpreted as compatible with models of balancing selection acting on temperamental traits within the population. Rather than reflecting a single pathogenic pathway, the presence of numerous common variants with small individual effects may represent the genetic substrate of behavioral diversity, including traits related to energy regulation, novelty seeking, creativity, and social drive. In this context, the partial genetic overlap observed between bipolar disorder and other psychiatric conditions may not solely indicate shared vulnerability, but could also reflect the evolutionary maintenance of variation in psychological and behavioral traits that may confer advantages in certain environmental or social contexts. This may reflect a shared genetic vulnerability contributing to both bipolar disorder and schizophrenia (34, 35). Furthermore, alternative hypotheses have also been proposed (36, 37).

The large number of genetic variants associated with bipolar disorder, each contributing a small effect, highlights the complex and heterogeneous biological architecture of the condition (38).

1. Given that the disorder is defined on a descriptive syndromic basis, it is possible that different etiopathogenetic pathways lead to similar symptoms (39).
2. Within these different etiopathogenetic pathways, it is possible that the emergence of epiphenomena worthy of clinical relevance is also the result of the encounter between predisposing factors, including genetics, that may become pathological in interaction with the environment, especially in the most vulnerable stages of life (40).

This perspective is also linked to the evidence that the prevalence of mood disorders is at least stable in the population over time, but with some evidence of an increase in the last decades in the United States (41), New South Wales (42), and Korea (43).

**Figure 1:**
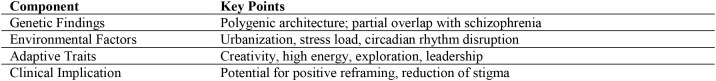
Evolutionary-adaptive hypothesis of bipolar disorder: genetic, environmental, and functional aspects. This schematic representation illustrates a heuristic conceptual model integrating genetic vulnerability, environmental influences, and temperamental traits associated with the bipolar spectrum. It is intended as an interpretative framework to facilitate discussion of potential evolutionary mechanisms and should not be considered an empirically validated causal model.

Multiple studies hypothesize that the persistence of prevalence may relate to the fact that some aspects of bipolar spectrum disorders may offer advantages in areas such as creativity and inventiveness (44)(**Figure 1**). It is important to distinguish between different levels of expression within the bipolar spectrum when discussing potential adaptive traits. Evidence suggesting associations with creativity, novelty seeking, leadership, or resilience most often refers to subclinical bipolar spectrum traits or temperamental characteristics observed in unaffected relatives of individuals with bipolar disorder. In contrast, the fully expressed clinical disorder is typically associated with significant functional impairment, mood instability, and increased morbidity. Therefore, the possible adaptive value proposed in the evolutionary framework should be understood primarily in relation to temperamental or subclinical traits that may exist along a continuum of vulnerability, rather than to the severe clinical manifestations of bipolar disorder. Importantly, this perspective does not imply that acute manic or depressive episodes are adaptive or beneficial, as these episodes are frequently associated with substantial clinical impairment and risk.

It emerges that divergent thinking, often present in these clinical pictures, allows for a good level of cognitive ability with activation of the associative network and creative ideation, conferring an advantageous trait in areas such as writing (19). Further supporting the hypothesis of adaptive advantages, studies have found that features of the bipolar spectrum, particularly hyperthymic traits, are more frequently observed among students in artistic disciplines and individuals engaged in highly creative fields (45, 46). Similarly, elevated levels of risk-taking behavior, often associated with hypomanic temperamental features, have been reported among elite athletes participating in extreme and high-risk sports disciplines (45–47). These findings suggest that creativity, novelty seeking, and resilience under stress may have provided significant evolutionary advantages in exploration, innovation, and social leadership (47). Mixed presentations in bipolar disorder illustrate how the boundary between temperamental traits and clinically impairing mood dysregulation can become blurred, highlighting the dimensional nature of bipolar phenomenology and the importance of careful diagnostic and therapeutic management (48). A hyperthymic, social, and energetic temperament may bring advantages in leadership and exploration. At the same time, it is important to recognize that the expression of bipolar spectrum traits is highly heterogeneous and strongly influenced by contextual moderators. Cultural environments, occupational demands, and social structures may shape whether characteristics such as elevated energy, novelty seeking, creativity, or risk-taking are expressed in adaptive or maladaptive ways. In certain contexts, such as creative professions, leadership roles, or exploratory environments, these traits may facilitate innovation, productivity, and resilience under stress. In other contexts, however, the same characteristics may contribute to functional difficulties, interpersonal conflict, or clinical destabilization (23, 49). This context-dependent variability highlights the importance of considering environmental and sociocultural factors when interpreting the potential adaptive dimensions of bipolar spectrum traits (49). In light of these considerations, it is hypothesized that subclinical forms of BD may have some adaptive advantage (50). Otherwise, it might be expected that a disorder associated with substantial functional impairment and reduced life expectancy would gradually decrease in frequency over evolutionary time, unless counterbalanced by mechanisms such as polygenic architecture, pleiotropic effects, or potential adaptive advantages linked to related temperamental traits (23, 51). Recognizing these traits as potential adaptive advantages rather than solely pathological expressions may help foster more nuanced societal views, potentially mitigating the stigma that often surrounds individuals diagnosed with BD.

In recent decades, large segments of the population have gradually shifted to a limited number of cities with millions of inhabitants, with markedly stressful social and life rhythms characterized by considerable demands on vital energy (19). The load resulting from daily lifestyle, actualized by social changes, increased business travel, noise and light pollution, and changes in eating habits, circadian and social rhythms (52), interacts with human metabolism (53–56).

Based on ethological and genetic evaluations of primates, hyperactive individuals may develop better stress adaptation (57). The literature proposes that 5-10% of young rhesus monkeys observed in the wild growing up in herds manifest hyperactivity and aggression under even moderate stress situations, showing altered central serotonin metabolism (58).

The rapidity of these social changes brought about by globalization has thus entailed changes in adaptation and communication (59). Exploratory and aggressive traits may be adaptive in marked stress due to gene-environment interactions. In contrast, in low-stress situations, the social network may perceive the aggressive trait as pathological by not turning out to be adaptive. This view thus has a more sustainable basis than hypotheses in which BD is viewed solely as an epiphenomenon of an altered genetic profile (60).

It seems reasonable to hypothesize that hyperactivity and increased energy level, potentially hypomanic elements, may be an advantage in social functioning (61, 62). It also emerges that social skills, exploratory and risk-taking behavior, problem-solving ability, and creativity are often improved among family members of people with BD, that is, in relatives without a BD diagnosis and even well-adjusted relatives (63, 64).

Furthermore, while the mild to moderate expression of shared vulnerability traits may have some advantage concerning greater creativity, the more obvious forms of pathology characterized by poor judgment and greater impulsivity appear to detract from good social performance (65, 66).

Current clinical therapeutic practice in bipolar disorder has traditionally focused primarily on symptom stabilization and relapse prevention, sometimes leaving less room for approaches aimed at integrating patients’ strengths, functional capacities, and individual life goals. It should likewise be noted that multiple aspects of creative occupations can have an exacerbating or stimulating effect on symptomatology, in particular, increased stress, use of psychotropic substances and alcohol, and dysregulated schedules (63). Nonetheless, the positive aspects of stimulated creativity may include a higher level of contentment and improved social functioning; thus, it is evident that the activation of creative functionality and its expression at the occupational level could be a challenge for the clinical management practice of the individual with BD (67, 68).

The literature of recent decades provides consistent evidence about a familial link between manifestations of BD and creativity. Still, more knowledge must be gained to support the hypothesis about a net link between BD and the creative process. However, at the same time, it has been ascertained that a moderately elevated mood can increase the fluidity of thinking, particularly in its divergent form. In contrast, a highly elevated mood can antagonize the creative realization process (69–72). In defining these aspects, therefore, it is evident that shared vulnerabilities reflecting both a predisposition to creativity and a genetic risk for the disease may be considered candidates for adaptive traits according to a balancing selection suitable to allow the persistence of BD in the population despite the inherent disadvantages arising from the pathological picture. This interpretation is consistent with broader evolutionary models suggesting that behavioral diversity within human populations may be maintained through mechanisms of balancing selection. According to these models, temperamental traits related to increased energy, novelty seeking, and risk-taking may confer advantages in certain environmental or social contexts, even if their extreme expressions increase vulnerability to psychiatric conditions. From this perspective, bipolar spectrum traits may represent the upper tail of normally distributed adaptive behavioral strategies that have historically contributed to exploration, innovation, and leadership within human groups (73, 74). Considering these aspects from a genetic perspective may further elucidate the biological mechanisms underlying disease risk. It may also contribute to identifying novel targets for both psychosocial and pharmacological interventions (75). The debate can also be observed in the literature regarding the possible identification of a specific dysregulation of mood, energy, and social rhythms syndrome, characterized by a high degree of personal distress with reduced social functioning, which could be the result of a nonspecific response to environmental hyper-stress under conditions of increased individual, genetic, social, and clinical vulnerability (19, 76). Furthermore, growing evidence suggests that disturbances in biological rhythms, including sleep-wake cycles and activity patterns, are a core feature of bipolar disorder. Studies have consistently shown a higher prevalence of the evening chronotype among individuals with bipolar spectrum disorders, characterized by a preference for later sleep and activity times. In a society predominantly oriented toward morning activities and schedules, this misalignment may exacerbate stress, contribute to social and occupational dysfunction, and increase vulnerability to mood episodes (77). Such positive reframing of creative abilities may serve as a key component in counteracting stigma and promoting a strengths-based model of care for individuals at risk or diagnosed with BD.

It is important to emphasize that the evolutionary perspective discussed in this article should be interpreted primarily as a heuristic framework rather than a definitive explanatory model. While several empirical findings support the association between bipolar spectrum traits and domains such as creativity, novelty seeking, and adaptive stress responses, the hypothesis that these traits may have been maintained through evolutionary processes remains partly speculative. Consequently, the evidence presented here should be understood as an attempt to integrate established findings with emerging theoretical interpretations, with the aim of stimulating further interdisciplinary research on the biological and psychosocial mechanisms underlying bipolar spectrum conditions.

The current approach to BD identifies adaptive potential as a focus of psychosocial treatment and drug therapy (78). This interpretation of clinical and social management could also decrease discrimination and stigma toward people suffering from BD (79–82).

Beyond its conceptual and stigma-reducing implications, the evolutionary-adaptive framework may also contribute to clinical reasoning in several ways. Recognizing that certain temperamental features associated with the bipolar spectrum, such as elevated energy, creativity, novelty seeking, and social drive, may represent amplified expressions of traits that can be advantageous in specific contexts may encourage clinicians to adopt a more nuanced and strengths-based perspective. In psychosocial practice, this approach may support interventions aimed at helping individuals channel these traits into structured and adaptive activities, while simultaneously promoting strategies to regulate sleep–wake cycles, stress exposure, and emotional reactivity. Such a perspective does not replace established evidence-based pharmacological and psychosocial treatments but may complement them by fostering more personalized approaches that integrate symptom management with the promotion of functional strengths. From a clinical perspective, this evolutionary interpretation should not be considered a direct guide for treatment decisions; rather, it may contribute to a broader conceptual framework that encourages clinicians to recognize both the vulnerabilities and the potential functional strengths associated with bipolar spectrum temperamental traits.

In addition, promoting an understanding of bipolar disorder as a condition potentially rooted in adaptive traits offers valuable avenues for reducing stigma. Nevertheless, it is important to acknowledge the potential risk of misinterpretation when discussing adaptive or strengths-related aspects of bipolar spectrum traits. Such perspectives should not be interpreted as romanticizing bipolar disorder or minimizing the significant disability, relapse risk, and clinical burden associated with the condition. Rather, a strengths-based reframing should be understood as a complementary perspective that coexists with a realistic clinical recognition of the challenges and vulnerabilities experienced by many individuals with bipolar disorder.

Such a reframing could lead to psychosocial strategies that not only aim at symptom control but also at enhancing patients’ strengths, ultimately contributing to better clinical outcomes and improved societal acceptance. At the same time, it is essential to acknowledge the limitations of the evolutionary perspective proposed in this article. Bipolar disorder remains a severe psychiatric condition associated with significant functional impairment, high relapse rates, and increased risk of suicide and premature mortality. Therefore, the hypothesis that certain bipolar spectrum traits may have historically conferred adaptive advantages should not be interpreted as minimizing the profound suffering and clinical burden experienced by many individuals with the disorder. Rather, this framework should be viewed as a complementary perspective that may help contextualize certain temperamental features within broader evolutionary and psychosocial processes. In clinical practice, such an interpretation may contribute to a more balanced and stigma-reducing approach, while maintaining a clear focus on early diagnosis, evidence-based treatment, and the prevention of adverse outcomes. In conclusion, although BD is still currently a relevant issue in health care, potentially causing marked disability, the possibility emerges from the literature that it represents an adaptive advantage in situations of marked stress, suggesting how individualized treatment strategies aimed at maintaining such positive aspects while limiting disadvantageous manifestations could lead to improved outcomes.

In summary, considering bipolar disorder through an evolutionary-informed perspective may offer a complementary framework for integrating genetic vulnerability, temperamental traits, and environmental influences. Such an approach may have several implications: clinically, it may encourage more nuanced and strengths-informed perspectives on bipolar spectrum traits; societally, it may contribute to reducing stigma by challenging exclusively deficit-based narratives; and scientifically, it may stimulate further interdisciplinary research on the evolutionary and biological mechanisms underlying mood dysregulation. At the same time, this perspective must be interpreted with caution and should not be understood as minimizing the significant clinical burden associated with bipolar disorder. Rather, it should be viewed as a heuristic framework that may enrich current models while remaining fully compatible with established clinical and neurobiological knowledge.

## References

[1] McIntyre RS Berk M Brietzke E Goldstein BI López-Jaramillo C Kessing LV . Bipolar disorders. Lancet. (2020) 396:1841–56. doi: 10.1016/S0140-6736(20)31544-0, PMID: 33278937

[2] Goldberg JF Perlis RH Bowden CL Thase ME Miklowitz DJ Marangell LB . Manic symptoms during depressive episodes in 1,380 patients with bipolar disorder: findings from the STEP-BD. Am J Psychiatry. (2009) 166:173–81. doi: 10.1176/appi.ajp.2008.08050746, PMID: 19122008 PMC10034853

[3] Vieta E Reinares M Rosa AR . Staging bipolar disorder. Neurotox Res. (2011) 19:279–85. doi: 10.1007/s12640-010-9197-8, PMID: 20461491

[4] Berk M Post R Ratheesh A Gliddon E Singh A Vieta E . Staging in bipolar disorder: from theoretical framework to clinical utility. World Psychiatry. (2017) 16:236–44. doi: 10.1002/wps.20441, PMID: 28941093 PMC5608827

[5] Kapczinski F Magalhães PVS Balanzá-Martinez V Dias VV Frangou S Gama CS . Staging systems in bipolar disorder: an International Society for Bipolar Disorders Task Force Report. Acta Psychiatr Scand. (2014) 130:354–63. doi: 10.1111/acps.12305, PMID: 24961757

[6] Carta MG Ouali U Perra A Ben Cheikh Ahmed A Boe L Aissa A . Living with bipolar disorder in the time of covid-19: biorhythms during the severe lockdown in Cagliari, Italy, and the moderate lockdown in Tunis, Tunisia. Front Psychiatry. (2021) 12:634765. doi: 10.3389/fpsyt.2021.634765, PMID: 33716829 PMC7943838

[7] Alloy LB Abramson LY Walshaw PD Cogswell A Grandin LD Hughes ME . Behavioral Approach System and Behavioral Inhibition System sensitivities and bipolar spectrum disorders: prospective prediction of bipolar mood episodes. Bipolar Disord. (2008) 10:310–22. doi: 10.1111/j.1399-5618.2007.00547.x, PMID: 18271911

[8] Rosenblat JD McIntyre RS . Treatment-resistant bipolar disorder: a review of therapeutic options and clinical considerations. J Affect Disord. (2020) 276:121–31. doi: 10.1016/j.jad.2020.07.040, PMID: 32871664

[9] Fornaro M Carvalho AF Fusco A Anastasia A Solmi M Berk M . The concept and management of acute episodes of treatment-resistant bipolar disorder: a systematic review and exploratory meta-analysis of randomized controlled trials. J Affect Disord. (2020) 276:970–83. doi: 10.1016/j.jad.2020.07.109, PMID: 32750614

[10] Jakobsen P Côté-Allard U Riegler MA Stabell LA Stautland A Nordgreen T . Early warning signals observed in motor activity preceding mood state change in bipolar disorder. Bipolar Disord. (2024) 26:468–78. doi: 10.1111/bdi.13430, PMID: 38639725

[11] Tarricone I D’Andrea G Storbini V Braca M Ferrari S Reggianini C . First-episode psychosis and migration in Italy: results from a study in the Italian mental health services (Pep-Ita study). J Immigr Minor Health. (2021) 23:519–27. doi: 10.1007/s10903-021-01168-w, PMID: 33689115 PMC8068695

[12] McIntyre RS Alda M Baldessarini RJ Bauer M Berk M Correll CU . The clinical characterization of the adult patient with bipolar disorder aimed at personalization of management. World Psychiatry. (2022) 21:364–87. doi: 10.1002/wps.20997, PMID: 36073706 PMC9453915

[13] Meyer TD Perich T Jones SH Lee TMC . Bipolar and related disorders. In: A psychological approach to diagnosis: Using the ICD-11 as a framework. American Psychological Association, Washington, DC, US (2024). p. 117–36. doi: 10.1037/0000392-007, PMID:

[14] Silverman AM Dimick MK Barton JS Youngstrom EA Goldstein BI . Comparing symptoms of major depression in youth with confirmed versus suspected bipolar disorder. J Child Adolesc Psychopharmacol. (2024) 34:194–200. doi: 10.1089/cap.2023.0090, PMID: 38588580

[15] Baldessarini RJ Vieta E Calabrese JR Tohen M Bowden CL . Bipolar depression: overview and commentary. Harv Rev Psychiatry. (2010) 18:143. doi: 10.3109/10673221003747955, PMID: 20415631

[16] Hirschfeld RMA . Bipolar depression: the real challenge. Eur Neuropsychopharmacol. (2004) 14:S83–8. doi: 10.1016/j.euroneuro.2004.03.001, PMID: 15142612

[17] Fornaro M Anastasia A Novello S Fusco A Solmi M Monaco F . Incidence, prevalence and clinical correlates of antidepressant-emergent mania in bipolar depression: a systematic review and meta-analysis. Bipolar Disord. (2018) 20:195–227. doi: 10.1111/bdi.12612, PMID: 29441650

[18] Kessler RC Berglund P Demler O Jin R Merikangas KR Walters EE . Lifetime prevalence and age-of-onset distributions of DSM-IV disorders in the national comorbidity survey replication. Arch Gen Psychiatry. (2005) 62:593–602. doi: 10.1001/archpsyc.62.6.593, PMID: 15939837

[19] Carta MG Kalcev G Fornaro M Pinna S Gonzalez CIA Nardi AE . Does screening for bipolar disorders identify a “Dysregulation of mood, energy, and social rhythms syndrome” (DYMERS)? A heuristic working hypothesis. J Clin Med. (2023) 12:5162. doi: 10.3390/jcm12155162, PMID: 37568562 PMC10419483

[20] Taylor L Faraone SV Tsuang MT . Family, twin, and adoption studies of bipolar disease. Curr Psychiatry Rep. (2002) 4:130–3. doi: 10.1007/s11920-002-0046-1, PMID: 11914174

[21] Barnett JH Smoller JW . The genetics of bipolar disorder. Neuroscience. (2009) 164:331–43. doi: 10.1016/j.neuroscience.2009.03.080, PMID: 19358880 PMC3637882

[22] Kalcev G Scano A Orrù G Primavera D Cossu G Nardi AE . Is a Genetic Variant associated with Bipolar Disorder Frequent in People without Bipolar Disorder but with Characteristics of Hyperactivity and Novelty Seeking? Clin Pract Epidemiol Ment Health. (2023) 19:e174501792303280. doi: 10.2174/17450179-v19-e230419-2022-53, PMID: 37916199 PMC10351339

[23] Greenwood TA . Creativity and bipolar disorder: A shared genetic vulnerability. Annu Rev Clin Psychol. (2020) 16:239–64. doi: 10.1146/annurev-clinpsy-050718-095449, PMID: 32040337

[24] Scano A Orrù G Kalcev G Tusconi M Spada M Atzori L . Adaptive hyperactivity and biomarker exploration: insights from elders in the blue zone of sardinia. J Clin Med. (2024) 13:6451. doi: 10.3390/jcm13216451, PMID: 39518590 PMC11547069

[25] Sancassiani F Carta MG Primavera D Tusconi M Urban A Atzori L . The breathomics profile of volatile sulfur compounds in the bipolar spectrum, does it represent a potential tool for early diagnosis? J Clin Med. (2025) 14:2025. doi: 10.3390/jcm14062025, PMID: 40142833 PMC11942791

[26] Sklar P Gabriel SB McInnis MG Bennett P Lim Y-M Tsan G . Family-based association study of 76 candidate genes in bipolar disorder: BDNF is a potential risk locus. Mol Psychiatry. (2002) 7:579–93. doi: 10.1038/sj.mp.4001058, PMID: 12140781

[27] Stahl EA Breen G Forstner AJ McQuillin A Ripke S Trubetskoy V . Genome-wide association study identifies 30 loci associated with bipolar disorder. Nat Genet. (2019) 51:793–803. doi: 10.1038/s41588-019-0397-8, PMID: 31043756 PMC6956732

[28] Kalcev G Cossu G Preti A Littera MT Frau S Primavera D . Development and validation of the questionnaire for adaptive hyperactivity and goal achievement (AHGA). Clin Pract Epidemiol Ment Health. (2023) 19:e174501792303281. doi: 10.2174/17450179-v19-e230419-2022-50, PMID: 37916197 PMC10351347

[29] Perrier E Pompei F Ruberto G Vassos E Collier D Frangou S . Initial evidence for the role of CACNA1C on subcortical brain morphology in patients with bipolar disorder. Eur Psychiatry. (2011) 26:135–7. doi: 10.1016/j.eurpsy.2010.10.004, PMID: 21292451

[30] Bhat S Dao DT Terrillion CE Arad M Smith RJ Soldatov NM . *CACNA1C* (Cav1.2) in the pathophysiology of psychiatric disease. Prog Neurobiol. (2012) 99:1–14. doi: 10.1016/j.pneurobio.2012.06.001, PMID: 22705413 PMC3459072

[31] Zhang C Xiao X Li T Li M . Translational genomics and beyond in bipolar disorder. Mol Psychiatry. (2021) 26:186–202. doi: 10.1038/s41380-020-0782-9, PMID: 32424235

[32] Li H Zhou D-S Chang H Wang L Liu W Dai S-X . Interactome Analyses implicated *CAMK2A* in the genetic predisposition and pharmacological mechanism of Bipolar Disorder. J Psychiatr Res. (2019) 115:165–75. doi: 10.1016/j.jpsychires.2019.05.024, PMID: 31150948

[33] Muntané G Farré X Bosch E Martorell L Navarro A Vilella E . The shared genetic architecture of schizophrenia, bipolar disorder and lifespan. Hum Genet. (2021) 140:441–55. doi: 10.1007/s00439-020-02213-8, PMID: 32772156

[34] Zhao NO Topolski N Tusconi M Salarda EM Busby CW Lima CNNC . Blood-brain barrier dysfunction in bipolar disorder: Molecular mechanisms and clinical implications. Brain Behav Immun - Health. (2022) 21:100441. doi: 10.1016/j.bbih.2022.100441, PMID: 35308081 PMC8924633

[35] Tusconi M Fries GR . Chapter 11 - Neuroprogression in bipolar disorder. In: MaChado-Vieira R Soares JC , editors. Biomarkers in bipolar disorders. San Diego, CA, United States: Academic Press (2022). 167–89. doi: 10.1016/B978-0-12-821398-8.00009-6, PMID:

[36] Charney AW Mullins N Park YJ Xu J . On the diagnostic and neurobiological origins of bipolar disorder. Transl Psychiatry. (2020) 10:1–10. doi: 10.1038/s41398-020-0796-8, PMID: 32327632 PMC7181677

[37] Chaves C Dursun SM Tusconi M Hallak JEC . Neuroinflammation and schizophrenia – is there a link? Front Psychiatry. (2024) 15:1356975. doi: 10.3389/fpsyt.2024.1356975, PMID: 38389990 PMC10881867

[38] Gonda X Petschner P Eszlari N Baksa D Edes A Antal P . Genetic variants in major depressive disorder: From pathophysiology to therapy. Pharmacol Ther. (2019) 194:22–43. doi: 10.1016/j.pharmthera.2018.09.002, PMID: 30189291

[39] Xu F Su Y Wang X Zhang T Xie T Wang Y . Olink proteomics analysis uncovers inflammatory proteins in patients with different states of bipolar disorder. Int Immunopharmacol. (2024) 131:111816. doi: 10.1016/j.intimp.2024.111816, PMID: 38484669

[40] Post RM Leverich GS . The role of psychosocial stress in the onset and progression of bipolar disorder and its comorbidities: The need for earlier and alternative modes of therapeutic intervention. Dev Psychopathol. (2006) 18:1181–211. doi: 10.1017/S0954579406060573, PMID: 17064434

[41] Compton WM Conway KP Stinson FS Grant BF . Changes in the prevalence of major depression and comorbid substance use disorders in the United States between 1991–1992 and 2001–2002. Am J Psychiatry. (2006) 163:2141–7. doi: 10.1176/ajp.2006.163.12.2141, PMID: 17151166

[42] Goldney RD Eckert KA Hawthorne G Taylor AW . Changes in the prevalence of major depression in an Australian community sample between 1998 and 2008. Aust New Z J Psychiatry. (2010) 44:901–10. doi: 10.3109/00048674.2010.493502, PMID: 20932204

[43] Lee J Kim H Hong JP Cho S-J Lee J-Y Jeon HJ . Trends in the prevalence of major depressive disorder by sociodemographic factors in Korea: results from nationwide general population surveys in 2001, 2006, and 2011. J Korean Med Sci. (2021) 36:e244. doi: 10.3346/jkms.2021.36.e244, PMID: 34636501 PMC8506416

[44] Carta MG Fornaro M Primavera D Nardi AE Karam E . Dysregulation of mood, energy, and social rhythms syndrome (DYMERS): A working hypothesis. J Public Health Res. (2024) 13:22799036241248022. doi: 10.1177/22799036241248022, PMID: 38680762 PMC11047225

[45] Kyaga S Lichtenstein P Boman M Hultman C Långström N Landén M . Creativity and mental disorder: Family study of 300–000 people with severe mental disorder. Br J Psychiatry. (2011) 199:373–9. doi: 10.1192/bjp.bp.110.085316, PMID: 21653945

[46] MacCabe JH Sariaslan A Almqvist C Lichtenstein P Larsson H Kyaga S . Artistic creativity and risk for schizophrenia, bipolar disorder and unipolar depression: a Swedish population-based case–control study and sib-pair analysis. Br J Psychiatry. (2018) 212:370–6. doi: 10.1192/bjp.2018.23, PMID: 29697041

[47] Siwek M Dudek D Drozdowicz K Jaeschke R Styczen K Arciszewska A . Temperamental dimensions of the TEMPS-A in male and female subjects engaging in extreme or/and high risk sports. J Affect Disord. (2015) 170:66–70. doi: 10.1016/j.jad.2014.08.036, PMID: 25233241

[48] Fornaro M Stubbs B De Berardis D Perna G Valchera A Veronese N . Atypical antipsychotics in the treatment of acute bipolar depression with mixed features: A systematic review and exploratory meta-analysis of placebo-controlled clinical trials. Int J Mol Sci. (2016) 17:241. doi: 10.3390/ijms17020241, PMID: 26891297 PMC4783972

[49] Jamison KR . Touched with fire: Manic-depressive illness and the artistic temperament. New York, NY, US: Free Press (1993). p. 370.

[50] Key FM Teixeira JC de Filippo C Andrés AM . Advantageous diversity maintained by balancing selection in humans. Curr Opin Genet Dev. (2014) 29:45–51. doi: 10.1016/j.gde.2014.08.001, PMID: 25173959

[51] Vosburg SK . The effects of positive and negative mood on divergent-thinking performance. Creat Res J. (1998) 11:165–72. doi: 10.1207/s15326934crj1102_6, PMID: 38329648

[52] Primavera D Aviles Gonzalez CI Romano F Kalcev G Pinna S Minerba L . Does the response to a stressful condition in older adults with life rhythm dysregulations provide evidence of the existence of the “Dysregulation of mood, energy, and social rhythms syndrome”? Healthcare. (2024) 12:87. doi: 10.3390/healthcare12010087, PMID: 38200993 PMC10778618

[53] Carta MG Aguglia E Caraci F Dell’Osso L Di Sciascio G Drago F . Quality of life and Urban / Rural living: preliminary results of a community survey in Italy. Clin Pract Epidemiol Ment Health. (2012) 8:169–74. doi: 10.2174/1745017901208010169, PMID: 23248678 PMC3522089

[54] Mura G Rocha NBF Helmich I Budde H MaChado S Wegner M . Physical activity interventions in schools for improving lifestyle in European countries. Clin Pract Epidemiol Ment Health. (2021) 17:77–101. doi: 10.2174/1745017901511010077, PMID: 25834629 PMC4378026

[55] Carta MG Cossu G Pintus E Zaccheddu R Callia O Conti G . Moderate exercise improves cognitive function in healthy elderly people: results of a randomized controlled trial. Clin Pract Epidemiol Ment Health. (2021) 17:75–80. doi: 10.2174/1745017902117010075, PMID: 34733346 PMC8493830

[56] Carta M Preti A Akiskal H . Coping with the new era: noise and light pollution, hperactivity and steroid hormones. Towards an evolutionary view of bipolar disorders. Clin Pract Epidemiol Ment Health. (2018) 14:33–6. doi: 10.2174/1745017901814010033, PMID: 29541149 PMC5838624

[57] Suomi SJ . Gene-environment interactions and the neurobiology of social conflict. Ann N Y Acad Sci. (2003) 1008:132–9. doi: 10.1196/annals.1301.014, PMID: 14998879

[58] Carta MG Moro MF Piras M Ledda V Prina E Stocchino S . Megacities, migration and an evolutionary approach to bipolar disorder: a study of Sardinian immigrants in Latin America. Braz J Psychiatry. (2019) 42:63–7. doi: 10.1590/1516-4446-2018-0338, PMID: 31269095 PMC6986479

[59] Moussaoui D . Migration, globalization, and mental health. In: Moussaoui D Bhugra D Tribe R Ventriglio A , editors. Mental Health, Mental Illness and Migration. Springer, Singapore (2021). p. 15–25. doi: 10.1007/978-981-10-2366-8_2, PMID:

[60] Carta MG Nardi AE Pinna S Cossu G Gureje O . Multidisciplinary contributions towards an evolutive interpretation of bipolar disorders: could it be the pathological drift of a potentially adaptive condition? Braz J Psychiatry. (2023) 45:366–72. doi: 10.47626/1516-4446-2023-3154, PMID: 37307284 PMC10668319

[61] Feist GJ . A meta-analysis of personality in scientific and artistic creativity. Pers Soc Psychol Rev. (1998) 2:290–309. doi: 10.1207/s15327957pspr0204_5, PMID: 15647135

[62] Johnson SL Eisner LR Carver CS . Elevated expectancies among persons diagnosed with bipolar disorder. Br J Clin Psychol. (2009) 48:217–22. doi: 10.1348/014466509X414655, PMID: 19254445 PMC2847483

[63] Murray G Johnson SL . The clinical significance of creativity in bipolar disorder. Clin Psychol Rev. (2010) 30:721–32. doi: 10.1016/j.cpr.2010.05.006, PMID: 20579791 PMC3409641

[64] Sass LA . Schizophrenia, modernism, and the “Creative imagination”: on creativity and psychopathology. Creat Res J. (2001) 13:55–74. doi: 10.1207/S15326934CRJ1301_7, PMID: 33486653

[65] Johnson SL Murray G Fredrickson B Youngstrom EA Hinshaw S Bass JM . Creativity and bipolar disorder: Touched by fire or burning with questions? Clin Psychol Rev. (2012) 32:1–12. doi: 10.1016/j.cpr.2011.10.001, PMID: 22088366 PMC3409646

[66] Peluso MAM Hatch JP Glahn DC Monkul ES Sanches M Najt P . Trait impulsivity in patients with mood disorders. J Affect Disord. (2007) 100:227–31. doi: 10.1016/j.jad.2006.09.037, PMID: 17097740

[67] Murray G Suto M Hole R Hale S Amari E Michalak EE . Self-management strategies used by ‘high functioning’ individuals with bipolar disorder: from research to clinical practice. Clin Psychol Psychother. (2011) 18:95–109. doi: 10.1002/cpp.710, PMID: 20572206

[68] Murnane EL Cosley D Chang P Guha S Frank E Gay G . Self-monitoring practices, attitudes, and needs of individuals with bipolar disorder: implications for the design of technologies to manage mental health. J Am Med Inform Assoc. (2016) 23:477–84. doi: 10.1093/jamia/ocv165, PMID: 26911822 PMC11740754

[69] Srivastava S Ketter TA . The link between bipolar disorders and creativity: evidence from personality and temperament studies. Curr Psychiatry Rep. (2010) 12:522–30. doi: 10.1007/s11920-010-0159-x, PMID: 20936438

[70] Carta MG Kalcev G Scano A Primavera D Orrù G Gureye O . Is bipolar disorder the consequence of a genetic weakness or not having correctly used a potential adaptive condition? Brain Sci. (2023) 13:16. doi: 10.3390/brainsci13010016, PMID: 36671999 PMC9856125

[71] Okide CC Eseadi C Ezenwaji IO Ede MO Igbo RO Koledoye UL . Effect of a critical thinking intervention on stress management among undergraduates of adult education and extramural studies programs. Med (Baltimore). (2020) 99:e21697. doi: 10.1097/MD.0000000000021697, PMID: 32871885 PMC7458214

[72] Park E-H Jung MH . The impact of major depressive disorder on adaptive function: A retrospective observational study. Med (Baltimore). (2019) 98:e18515. doi: 10.1097/MD.0000000000018515, PMID: 31876742 PMC6946334

[73] Nettle D Bateson M . The evolutionary origins of mood and its disorders. Curr Biol. (2012) 22:R712–21. doi: 10.1016/j.cub.2012.06.020, PMID: 22975002

[74] Crespi B . Evolutionary genetics of affective disorders. In: Understanding Depression: A Translational Approach. Oxford University Press, Oxford (2009). p. 37–54.

[75] Burkhardt E Pfennig A Breitling G Pfeiffer S Sauer C Bechdolf A . Creativity in persons at-risk for bipolar disorder—A pilot study. Early Interv Psychiatry. (2019) 13:1165–72. doi: 10.1111/eip.12748, PMID: 30302918

[76] Carta MG Kalcev G Scano A Pinna S Gonzalez CIA Nardi AE . Screening, genetic variants, and bipolar disorders: can useful hypotheses arise from the sum of partial failures? Clin Pract. (2023) 13:853–62. doi: 10.3390/clinpract13040077, PMID: 37623258 PMC10453758

[77] Selvi Y Adem A Abdullah A Murat B Fatih S . Chronotype differences in suicidal behavior and impulsivity among suicide attempters. Chronobiol Int. (2011) 28:170–5. doi: 10.3109/07420528.2010.535938, PMID: 21231879

[78] Sancassiani F Lecca ME Pintus E Moro MF Caria R Minerba L . Could an innovative training program including contact sports and counseling help young people with traits of psychopathy and A history of school dropout? Clin Pract Epidemiol Ment Health. (2019) 15:49–57. doi: 10.2174/1745017901915010049, PMID: 31043997 PMC6463421

[79] Carta MG Colom F Erfurth A Fornaro M Grunze H Hantouche E . In memory of hagop akiskal. Clin Pract Epidemiol Ment Health. (2021) 17:48–51. doi: 10.2174/1745017902117010048, PMID: 34249138 PMC8227446

[80] Kovess-Masfety V Pilowsky DJ Goelitz D Kuijpers R Otten R Moro MF . Suicidal ideation and mental health disorders in young school children across Europe. J Affect Disord. (2015) 177:28–35. doi: 10.1016/j.jad.2015.02.008, PMID: 25745832

[81] Hardoy MC Serra M Carta MG Contu P Pisu MG Biggio G . Increased neuroactive steroid concentrations in women with bipolar disorder or major depressive disorder. J Clin Psychopharmacol. (2006) 26:379. doi: 10.1097/01.jcp.0000229483.52955.ec, PMID: 16855455

[82] Carta MG Angst J . Screening for bipolar disorders: A public health issue. J Affect Disord. (2016) 205:139–43. doi: 10.1016/j.jad.2016.03.072, PMID: 27442457

